# Metformin alters skeletal muscle transcriptome adaptations to resistance training in older adults

**DOI:** 10.18632/aging.104096

**Published:** 2020-10-18

**Authors:** Ameya S. Kulkarni, Bailey D. Peck, R. Grace Walton, Philip A. Kern, Jessica C. Mar, Samuel T. Windham, Marcas M. Bamman, Nir Barzilai, Charlotte A. Peterson

**Affiliations:** 1 Institute for Aging Research, Albert Einstein College of Medicine, Bronx, NY 10461, USA; 2 Department of Medicine, Division of Endocrinology, Albert Einstein College of Medicine, Bronx, NY 10461, USA; 3 Center for Muscle Biology, College of Health Sciences, University of Kentucky, Lexington, KY 40504, USA; 4 Department of Internal Medicine, Division of Endocrinology, and the Barnstable Brown Diabetes and Obesity Center, University of Kentucky, Lexington, KY 40504, USA; 5 Australian Institute for Bioengineering and Nanotechnology, University of Queensland, Brisbane, Queensland, Australia; 6 Center for Exercise Medicine, University of Alabama, Birmingham, AL 35233, USA; 7 Department of Surgery, School of Medicine, University of Alabama at Birmingham, AL 35233, USA; 8 Department of Cell, Development and Integrative Biology, School of Medicine, University of Alabama at Birmingham, AL 35233, USA; 9 Geriatric Research, Education, and Clinical Center, Birmingham VA Medical Center, Birmingham, AL 35233, USA

**Keywords:** aging, metformin, strength training, exercise-drug interaction, gene expression

## Abstract

Evidence from clinical trials and observational studies suggests that both progressive resistance exercise training (PRT) and metformin delay a variety of age-related morbidities. Previously, we completed a clinical trial testing the effects of 14 weeks of PRT + metformin (metPRT) compared to PRT with placebo (plaPRT) on muscle hypertrophy in older adults. We found that metformin blunted PRT-induced muscle hypertrophic response. To understand potential mechanisms underlying the inhibitory effect of metformin on PRT, we analyzed the muscle transcriptome in 23 metPRT and 24 plaPRT participants. PRT significantly increased expression of genes involved in extracellular matrix remodeling pathways, and downregulated RNA processing pathways in both groups, however, metformin attenuated the number of differentially expressed genes within these pathways compared to plaPRT. Pathway analysis showed that genes unique to metPRT modulated aging-relevant pathways, such as cellular senescence and autophagy. Differentially expressed genes from baseline biopsies in older adults compared to resting muscle from young volunteers were reduced following PRT in plaPRT and were further reduced in metPRT. We suggest that although metformin may blunt pathways induced by PRT to promote muscle hypertrophy, adjunctive metformin during PRT may have beneficial effects on aging-associated pathways in muscle from older adults.

## INTRODUCTION

Biological aging is characterized by a progressive decline of physiological and metabolic functions across multiple organ systems. One of the key phenotypes of aging is the loss of skeletal muscle mass, a condition known as age-associated muscle atrophy or sarcopenia [1–3]. This is usually accompanied by reduced strength, muscle quality and mobility, increased risk of frailty and falls, lack of endurance and poor physical performance [4]. In the western population, >40% of adults over the age of 60 have difficulties with daily activities such as walking or standing up from a chair etc. and >30% suffer from some kind of physical disability [5]. After the age of 60, striking changes occur in muscle physiology, corresponding to a decline in muscle mass and deterioration of muscle strength by ~2% each year [6]. The pathophysiology of age-related decline in muscle mass and function is multifactorial, including biological factors such as hormonal imbalance, neurodegeneration and motor neuron loss, increased inflammation and circulating cytokines, as well as environmental factors such as physical inactivity, inadequate nutritional intake and psychosocial factors [4, 5, 7]. Furthermore, age-associated changes in muscle metabolism, such as mitochondrial dysfunction and insulin resistance, can have severe implications in muscle homeostasis and regeneration [8, 9]. We have shown that progressive resistance exercise training (PRT) induces meaningful increases in muscle strength, power, and functional mobility, however, the hypertrophic response is, on average, reduced in old compared to young, especially within men [10]. The hypertrophic response to PRT is also highly variable in older adults (reviewed in [11]).

The use of pharmacological interventions to augment the effect of PRT on muscle hypertrophy in older adults has been proposed [12–15]. The biguanide metformin has been studied in the context of exercise capacity, quality of life and mood states, and metabolic adaptations, such as insulin production and clearance, oxidative stress and cardiometabolic health in older adults with prediabetes and Type II diabetes [16–18]. Metformin has been extensively used since the 1950s, as the first-line treatment against Type II diabetes and is one of the most commonly prescribed drugs in the world, either as a monotherapy or in combination with insulin or other anti-hyperglycemic agents [19]. Metformin directly inhibits mitochondrial enzymes including complex I [20], activates AMP-activated protein kinase (AMPK) [21], inhibits NF-κB signaling and specifically blunts the secretion of proinflammatory cytokines in macrophages [22, 23]. Due to its role beyond anti-hyperglycemia in modulating several fundamental pathways disrupted during chronic diseases and aging, repurposing metformin to treat cardiovascular diseases, cognitive decline, cancers, neurodegenerative diseases and ultimately, aging as a whole, has been proposed [24–29].

Two studies have shown that short-term metformin treatment and exercise do not exhibit synergy, but work in an antagonistic manner, where metformin attenuates the insulin sensitizing effect of exercise [30, 31]. Metformin has been shown to induce physiologically subtle decreases in peak aerobic capacity evidenced by a reduction in peak oxygen uptake, peak heart rate, peak ventilation, peak respiratory exchange ratio and exercise duration [32]. However, another study in prediabetic adults concluded that metformin in combination with aerobic and resistance exercise training lowered proinsulin concentrations and increased insulin clearance [33]. Recently, Konopka et al showed in older adults that metformin blunted aerobic exercise training-induced improvements in cardiorespiratory fitness, insulin sensitivity, and prevented the gain in muscle mitochondrial respiration capacity [34]. Thus, there may be a complex interplay between molecular mechanisms of exercise adaptations and pathways affected by metformin.

We originally undertook the MASTERS Trial to test the hypothesis that metformin would act synergistically with PRT to reduce the number of exercise non-responders regularly observed among older adults by reducing muscle inflammation. However, in the MASTERS Trial, metformin plus PRT inhibited muscle growth after 14 weeks of training [35]. In vitro, acute metformin treatment of human primary myotubes undergoing electrical pulse stimulation (exercise mimetic capable of inducing myotube hypertrophy) had a repressive effect on mTORC1 signaling, and upregulated AMPK phosphorylation [35]. Thus, metformin’s impairment of cardiorespiratory fitness and muscle mass gains in physically active older adults must be reconciled with benefits associated with health span.

The purpose of this study was to profile the muscle transcriptome response to PRT with or without adjunctive metformin in the MASTERS Trial, to identify potential mechanisms contributing to the blunted hypertrophic response in older adults [35]. Previously, global gene expression studies of human skeletal muscle aging have identified mitochondrial dysfunction, extracellular matrix organization, complement activation and ribosomal pathways comprising a differentially expressed aging signature [36, 37]. Effects of PRT on this signature, with and without metformin, may identify new intervention targets that counteract sarcopenia, and provide information on potential metformin-exercise interactions in muscle that are relevant to repurposing metformin to treat aging and age-related disorders.

## RESULTS

### Global gene expression changes in skeletal muscle with placebo + progressive resistance training (plaPRT) or metformin + progressive resistance training (metPRT)

**Figure 1:**
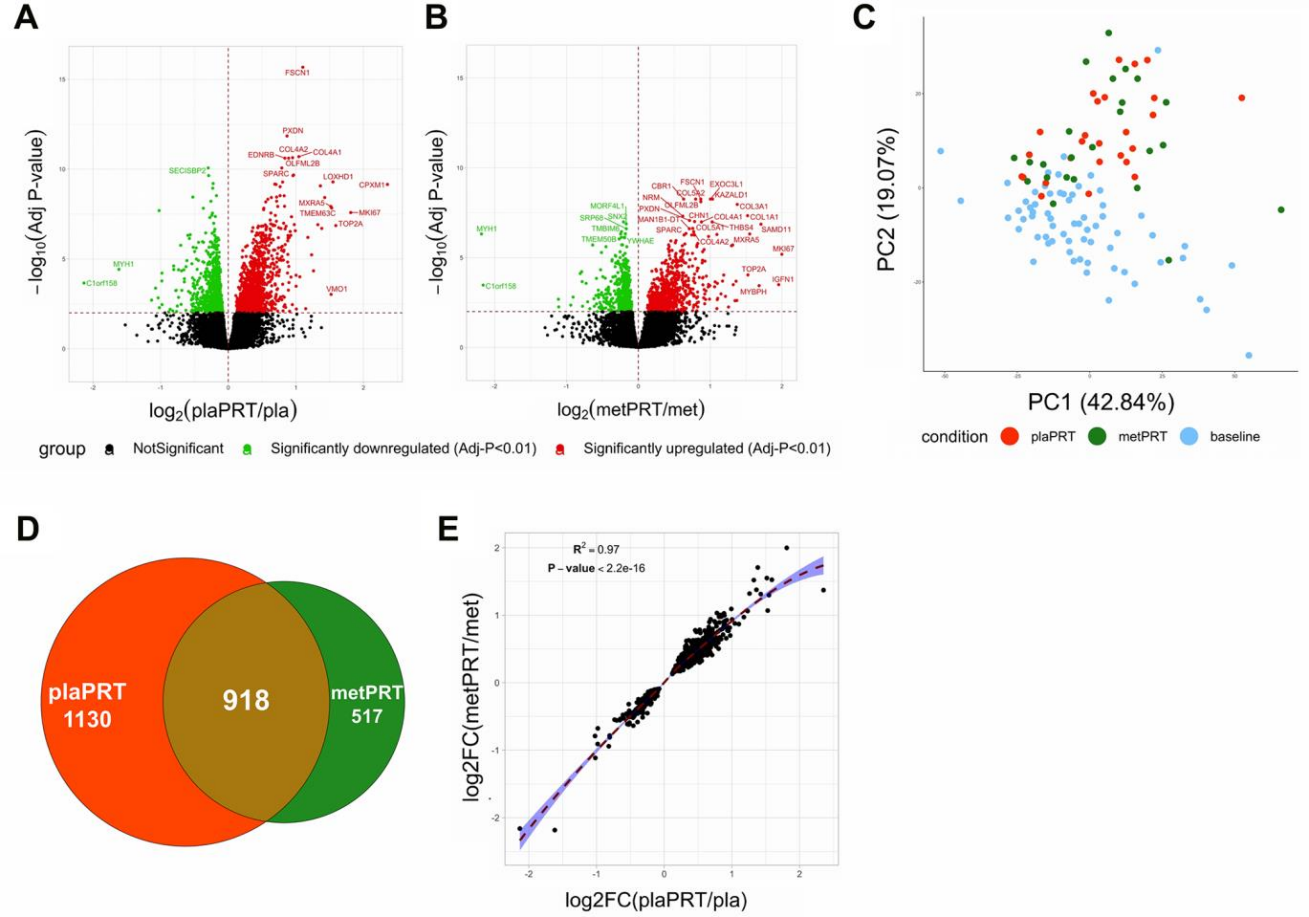
**Metformin blunts the global transcriptomic changes induced by PRT in human skeletal muscle.** (**A**) Volcano plot of 2048 DEG (q-value < 0.01) with plaPRT between 16 weeks and baseline; (**B**) Volcano plot of 1435 DEG (q-value < 0.01) with metPRT intervention between 16 weeks and baseline; (**C**) Principal component analysis on DEG shared between plaPRT (orange) and metPRT (green) compared to baseline (blue); (**D**) Venn diagram showing an overlap of DEG common between plaPRT and metPRT vs baseline; (**E**) Correlation plot between the fold changes of DEG common between plaPRT and metPRT.

Research participants were randomized in a double-blind fashion to placebo or metformin for 2 weeks followed by 14 weeks of PRT with continued drug treatment. Vastus lateralis biopsies were obtained at baseline (prior to drug treatment) and at 16 weeks (following 14 weeks of training) [35]. Fourteen weeks of placebo plus PRT (plaPRT) induced differential expression of 2048 genes (FDR-adj p-value < 0.01), with 1161 genes upregulated and 887 downregulated (Figure 1A). The combination of metformin and PRT (metPRT) resulted in differential expression of 1435 genes (FDR-adj p-value < 0.01), with 817 genes upregulated and 618 downregulated compared to baseline (Figure 1B). Principal component analysis (PCA) demonstrated a clear effect of PRT with a similar shift in the global gene expression profiles in both treatment groups (Figure 1C), with the Venn diagram illustrating an overlap of 918 genes (Figure 1D). The correlation between fold changes of the 918 common genes between the two groups was 0.97 (p-value < 2.2e-16), with no genes showing any anticorrelation (Figure 1E). Eleven hundred thirty genes were exclusively altered in plaPRT, while 517 genes were changed only in metPRT. Gene lists are included in Supplementary Table 1. We found no significant change in inflammatory pathway gene expression with either plaPRT or metPRT, which argues against our original hypothesis that metformin and PRT reduce muscle inflammation.

### Pathway overrepresentation within differentially expressed genes (DEG)

**Figure 2:**
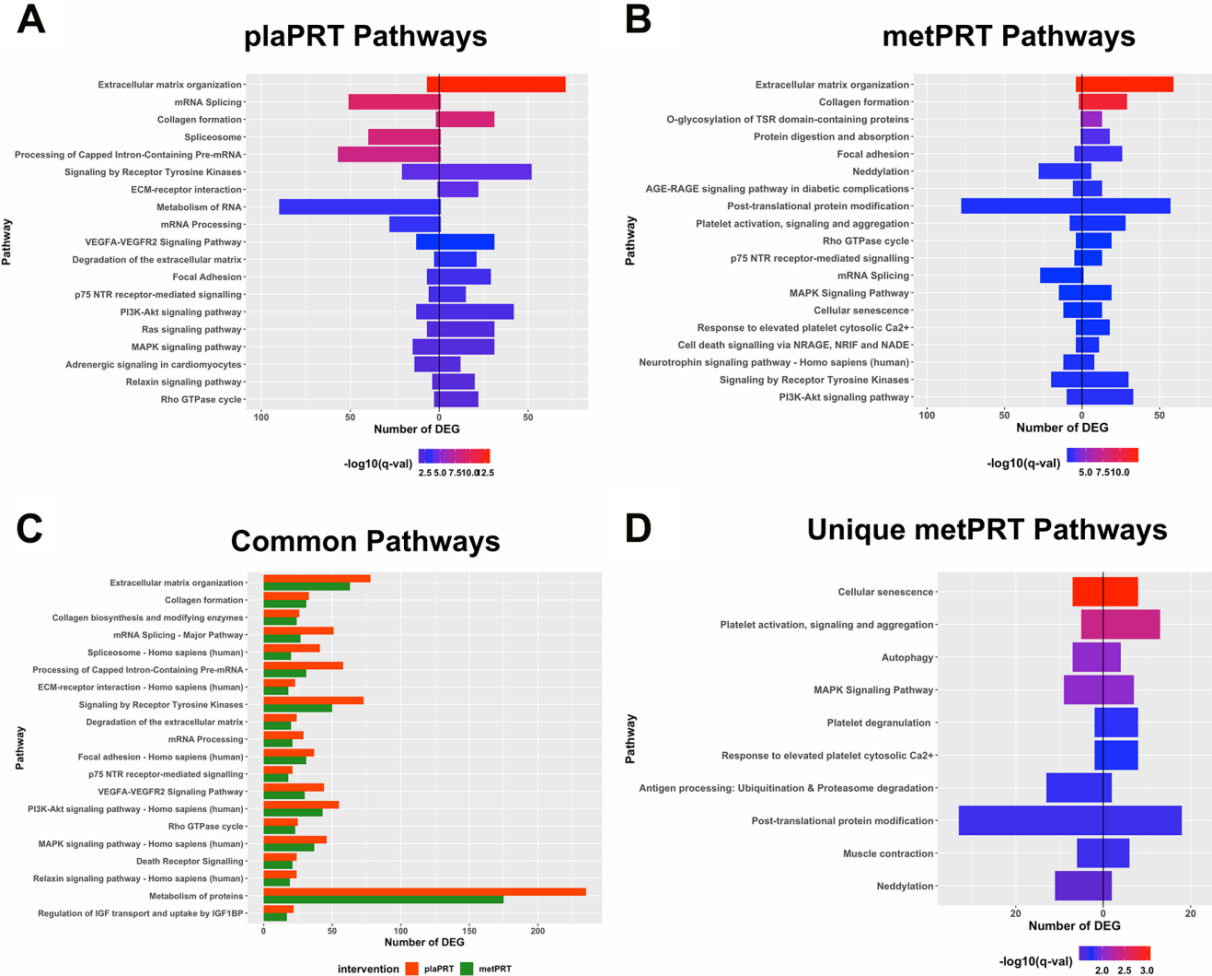
**Pathway overrepresentation analyses on differentially expressed genes (DEG).** (**A**) Pathways overrepresented in plaPRT-induced DEG with the length of the bar representing the number of DEG (upregulated genes to the right and downregulated genes to the left); (**B**) Pathways overrepresented in metPRT-induced DEG; (**C**) Common pathways overrepresented between the plaPRT (orange) and metPRT (green) groups; (**D**) Pathways overrepresented in the 517 DEG within the metPRT group that do not overlap with the DEG for the plaPRT group.

Both interventions modulated transcripts involved in anabolic cell signaling, extracellular matrix (ECM) organization, and RNA metabolism pathways, suggesting a conservation of these processes in response to PRT with metformin. The directionality of changes in gene expression demonstrate that ECM genes including collagen genes (COL1A1, COL3A1, COL4A1, COL6A2 etc.), ECM-receptor interaction genes, focal adhesion genes, and those encoding matrix metallopeptidases (MMP2, MMP11, MMP14) and laminin subunits were upregulated in both groups in response to training. On the other hand, genes belonging to mRNA splicing, RNA metabolism and post-transcriptional processing pathways were mostly downregulated. These included several nuclear ribonucleoproteins, serine and arginine rich splicing factors, RNA polymerase II subunit C, as well as pre-mRNA processing factors (Figures 2A, 2B, and Supplementary Table 2). However, the number of DEG was higher for all pathways in plaPRT than metPRT (Figure 2C).

Of the 517 genes that changed exclusively in the metPRT group, pathway analyses show overrepresentation of aging hallmarks including cellular senescence, and autophagy [38], as well as post-translational modification pathways, specifically neddylation and ubiquitination (Figure 2D and Supplementary Table 3). Our previous work with short-term metformin treatment alone showed improvement in aging-induced pathways through inhibition of mTORC1 regulated genes in skeletal muscle of older adults [39]; however, it is unclear how changes in gene expression observed here in metPRT might alter the older adult muscle phenotype.

### Reversal of the aging skeletal muscle transcriptome with PRT and metformin

**Figure 3:**
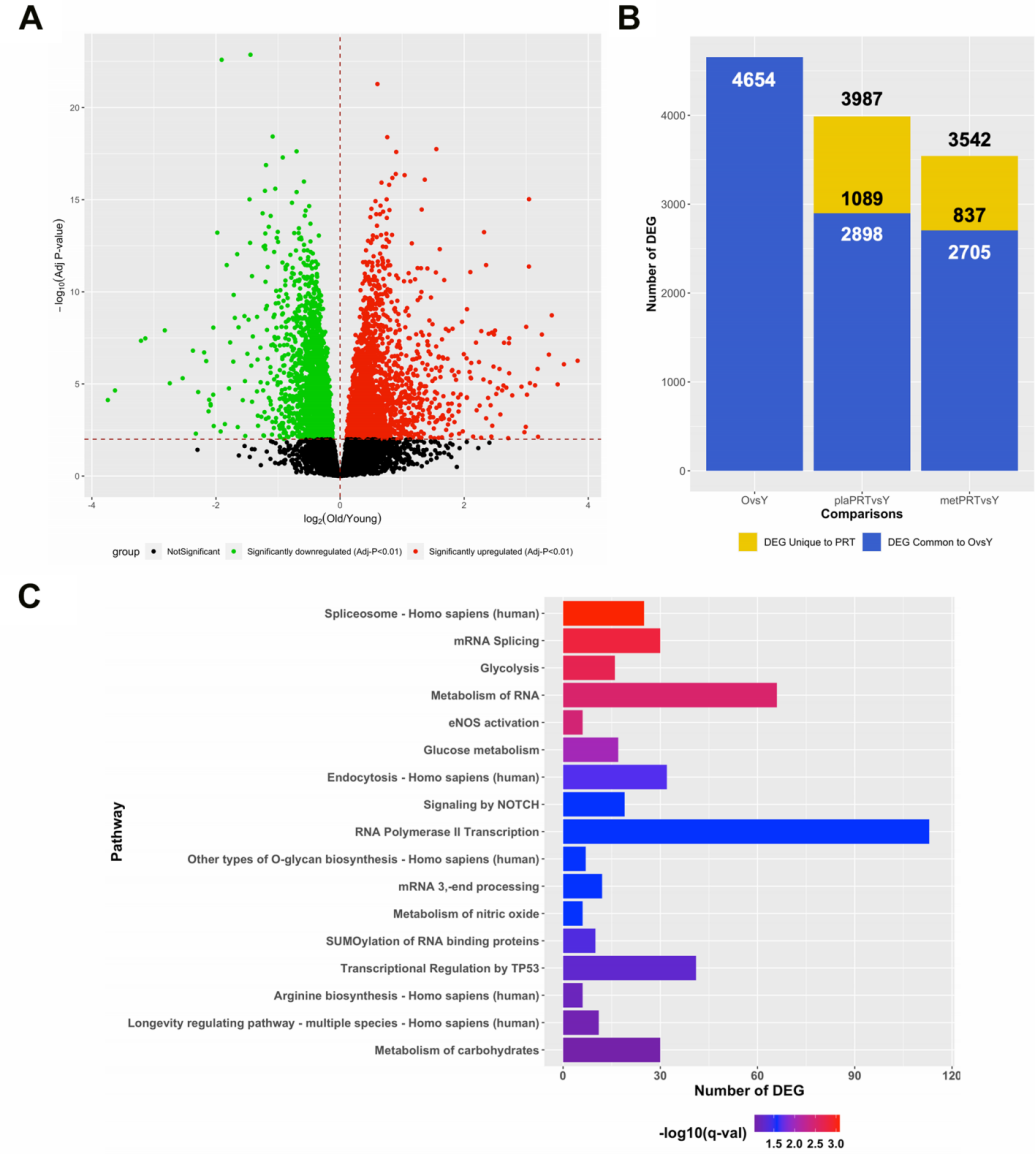
**PRT reverts aging transcriptome towards that of young resting muscle and the effects of metformin are additive.** (**A**) Volcano plot of 4654 DEG (q-value < 0.01) between young and old muscle at baseline; (**B**) Number of DEG observed when comparing young baseline skeletal muscle to old baseline skeletal muscle (4654 DEG) and young baseline to old 16 weeks with either plaPRT (3987 DEG) or metPRT (3542 DEG). Dark blue represents DEG common to throughout all time points and yellow represents those unique to week 16 time point in either plaPRT or metPRT; (**C**) Pathways overrepresented from those genes common to both groups following 14-weeks of PRT that were no different from young.

In lieu of our findings that metPRT appeared to affect pathways associated with aging, we performed RNA-sequencing on skeletal muscle biopsies from 21 young individuals (mean age 24, 11 females, 10 males, Supplementary Table 4) under resting conditions and compared their transcriptome to that of week 0 baseline biopsies from the older adult trial participants. Our analysis revealed that 4654 DEG were present when comparing baseline biopsies (FDR-adj p-value < 0.01) with 2446 upregulated and 2208 downregulated (Figure 3A and Supplementary Table 5). Following 14 weeks of PRT, the week 16 time point from plaPRT compared to young demonstrated a dramatic decrease in DEG that were previously observed at baseline (2898 DEG), with 1089 new DEG apparent following 14 weeks of plaPRT (Figure 3B and Supplementary Table 5). MetPRT further reduced the number of DEG compared to young muscle to 2705, however, the number of unique DEG that were present after 14 weeks of PRT was also lower compared to placebo (837 DEG), consistent with our initial findings that metformin blunts the overall transcriptomic response to PRT (Figure 3B and Supplementary Table 5). Young vs old baseline DEG that were lost following PRT in both groups (1483) include RNA splicing and numerous genes involved in longevity-associated pathways (Figure 3C and Supplementary Table 6). After identifying DEG between young and old muscle at baseline that were no longer differentially expressed at week 16 in both plaPRT and metPRT, we identified 466 DEG that returned to young expression levels only in metPRT. Pathway overrepresentation analysis showed that these genes were largely involved in metabolism, particularly lipid metabolism (Supplementary Table 7).

## DISCUSSION

In this study, we characterized the effect of metformin on the skeletal muscle transcriptomic response to PRT in older individuals from the MASTERS Trial [35]. Our results indicate that PRT induces substantial transcriptional changes in skeletal muscle with upregulation of genes involved in extracellular matrix (ECM) remodeling and downregulation of mRNA processing genes being the most affected. We also provide evidence that metformin alters skeletal muscle adaptations to PRT at the transcriptomic level, consistent with a decrease in physiological gains in response to PRT in lean body mass, and thigh muscle mass and area, as we have previously reported in this cohort [35]. Recently, the complex interaction between metformin and aerobic exercise training was described, showing that metformin also blunts improvements in physiological functions and mitochondrial adaptations otherwise promoted by aerobic exercise training in older adults [34]. Similarly, the present findings suggest that the key molecular cues underlying adaptations to PRT are attenuated by metformin. Nevertheless, we discovered gene sets differentially regulated by the combination of PRT and metformin that may promote health benefits separate from PRT-induced muscle hypertrophy.

Skeletal muscle ECM is crucial to force transmission, structural integrity and muscle stem cell dynamics [40, 41], and ECM remodeling is critical for muscle fiber growth [42]. A large number of genes associated with pathways involved in ECM composition and reorganization were upregulated in both plaPRT and metPRT groups, but to a lesser magnitude in metPRT, potentially contributing to the reduced growth response. Resistance exercise training in middle aged adults has been shown to upregulate collagens and metallopeptidases [43]. In particular, MMP2 plays an integral role in skeletal muscle hypertrophy by remodeling the ECM [44]. We found significant up-regulation of MMP2 mRNA in response to PRT in both groups, suggesting that metformin may not interfere with this specific effect of PRT that may contribute to muscle remodeling.

Genes belonging to mRNA splicing and post-transcriptional processing pathways were mostly downregulated in both groups. Aging is accompanied by a dysregulation in the splicing machinery including a rise in splicing factors, especially the heterogeneous nuclear ribonucleoproteins (hnRNPs) [45, 46]. We observed a systematic suppression of multiple hnRNPs and mRNAs encoding RNA processing and splicing factors, with down regulation of RNA processing pathways more prominent in plaPRT. Several studies have demonstrated that alternative splicing is highly enriched in energetically demanding tissues such as muscle and brain [47]. Upregulation of alternative splicing in skeletal muscle with aging may activate damage-response mechanisms at a time when energy becomes scarce [46]. Physical activity in older adults was shown to offset the changes in splicing machinery [48] and although the functional relevance has yet to be established, this effect may reduce the number of genes that are differentially spliced and the number of splicing errors that produce non-functional proteins that tend to increase with age [49].

To our knowledge only one other study has performed transcriptome analysis using microarrays to compare the effects of PRT in healthy older subjects to that of young resting skeletal muscle [50]. This seminal study illustrated that 596 genes were differentially expressed at baseline between young and old. Following 6 months of resistance exercise training a total of 179 of the 596 were no longer significantly different to that of young. Although our DEG lists are much larger, which is likely due to the greater number of older subjects at baseline between studies (65 vs 25), as well as our end point (26 plaPRT and 24 metPRT vs 14), we did find commonalities between gene lists including cell cycle inhibitors p21 and p15, as well as many metabolism related genes. Melov S et al. gene ontology analysis demonstrated that metabolic and mitochondrial function were largely impacted by aging and that resistance exercise training had a significant effect on reversing these age-related deficits [50]. Presently, we observed a similar response with PRT in both cohorts; however, adjunctive metformin further reduced the number of DEG between young and old, which appeared largely due to metformin’s effects on genes related to metabolism; pathway overrepresentation indicated a return to young gene expression patterns related to lipid metabolism preferentially in metPRT. In our primary findings of the trial, we reported an increase in AMPK/ACC phosphorylation in metPRT, that may affect lipogenesis [35]. The known inhibition of complex I by metformin [20] coupled with the increased energy demand of PRT may have led to compensatory activation of pathways involved in lipid metabolism that were previously dysregulated with age at baseline. However, it should be noted that outcomes of the MASTERS Trial showed that the PRT-induced decrease in low density muscle (which contains more intramyocellular lipid than normal density muscle) was similar between groups, whereas plaPRT gained significantly more normal density muscle area than metPRT [35]. Thus, the combined effects of metformin and exercise on muscle density and lipid content require further study.

A few limitations reduce the scope of our interpretation of the data, including the time between the last bout of exercise and tissue collection (3 days). It is possible that metformin inhibited mTOR signaling acutely after each exercise bout, impairing growth, but did not affect the new homeostasis following training. Although we reported down-regulation of mTOR-associated pathways in muscle following 6 weeks of metformin alone in a crossover study design in older adults [51], chronic effects of metformin on mTOR regulated pathways were less apparent within the context of exercise training. Another limitation acknowledged in our primary outcomes paper is the lack of a sedentary control group given metformin for 16 weeks. We attempted to extrapolate metformin-specific effects, but without a sedentary group, it is not possible to assess effects due to metformin or the combined effects of PRT + metformin on gene expression.

In conclusion, the blunted transcriptomic response to PRT in the presence of metformin is consistent with the blunted muscle hypertrophic growth response reported in the primary outcome of the MASTERS Trial [35]. However, specific effects of adjunctive metformin on the muscle transcriptomic response to PRT, separate from those related to muscle remodeling, may alter age-associated deficits in muscle metabolism to improve function. Metformin and PRT have beneficial effects on health that extend well-beyond skeletal muscle that should be considered, but a metformin-exercise interaction in muscle must be defined in more detail to inform recommending metformin for healthy, physically active older adults.

## MATERIALS AND METHODS

### Study design, participants and interventions

The Metformin to Augment Strength Training Effective Response in Seniors (MASTERS) Trial is a randomized, controlled, double blind trial comparing the effects of metformin versus placebo during a 14 week progressive resistance exercise training (PRT) intervention in healthy men and women ≥ 65 years of age. Participants were recruited at University of Kentucky and University of Alabama at Birmingham, UAB. The detailed study design [52] and participant characteristics [35] have been published previously.

Participants were randomized to receive either placebo or metformin for the duration of the trial. Subjects underwent a two week drug or placebo wash-in period prior to beginning PRT. Those who were randomized to metformin were titrated up to the target dose by taking 1 tablet per day (850 mg) for 7 days, followed by 2 tablets per day (1700 mg) for the remainder of the trial.

All study subjects underwent 14 weeks of PRT, supervised by trained personnel. We employed a variable intensity prescription across the three training days each week (high/low/high) based on the results of our previous dose-response trial which showed this prescription optimized strength and muscle mass gains in older adults [53]. Vastus lateralis muscle biopsies were obtained prior to drug initiation, after the 2 week wash-in period and 3 days after the final bout of training. Primary outcomes of the trial, muscle size and strength, have been reported [35]. Vastus lateralis muscle biopsies from young individuals matched for body mass index (Supplementary Table 4) were obtained through the Center for Muscle Biology at the University of Kentucky.

### Library preparation

Total RNA was isolated from baseline muscle biopsies in 37 plaPRT and 28 metPRT participants and from 16-week post-training muscle biopsies from 26 plaPRT and 24 metPRT participants (average age 71 years old). Of these, 24 plaPRT and 23 metPRT participants had biopsies at both timepoints. Additionally, total RNA was isolated from muscle biopsies in 21 young healthy donors (average age 24 years old). Approximately 35 mg of muscle was subjected to bead homogenization in Qiazol (Qiagen, Valencia, CA) and RNA purified using miRNeasy Mini Kits (Qiagen) and stored at -80° C. RNA content, integrity and purity were determined with a Nanodrop 2000 spectrophotometer (Thermo Fisher, Waltham, MA) and the 2100 Bioanalyzer (Agilent, Santa Clara, CA). A minimum RNA Integrity Number (RIN) of 6.5 was set for all samples.

### Sequencing, preprocessing and alignment

Total RNA was sequenced at Novogene Corporation, Chula Vista, CA on an Illumina HiSeq 4000 system, using a standard paired-end 150 bp (PE150) dual indexing protocol. The two sets of samples (University of Kentucky and University of Alabama), each containing samples from all timepoints of both placebo and metformin arm as well as young, were sequenced in different batches that were corrected for in the downstream analyses. Raw fastq sequence reads were passed through quality control using FastQC (0.11.4) [54] and the QC results were compiled for all samples using MultiQC (1.7) [55]. Due to adapter contamination, the raw fastq files were trimmed for adapter sequences, filtered for low quality reads and too short reads, using the default parameters in *fastp* (0.19.4) - an all-in-one preprocessing tool for fastq files [56]. After checking for QC using the same steps as before, RNA-Sequencing by Expectation Maximization- RSEM (1.3.0) in conjunction with the STAR aligner (2.6.1b) was used to align the raw reads to the GRCh38 primary assembly build of the reference human genome, with transcript annotations (gencode.v29.annotation.gtf) downloaded from GENCODE [57–59].

### Differential gene expression analysis

All statistical analysis for the gene expression data were carried out using the R statistical software (R-3.6.0). The raw counts were filtered for low expression using a counts-per-million (cpm) threshold of 0.6 (10/minimum library size) in at least 24 (number of samples in the smallest group of comparison). Since the principal component analyses revealed a distinct sequencing batch effect, the raw count data was corrected for it using *batch* as a covariate in the generalized linear model in limma (3.4.0) [60]. The corrected data using the *removeBatchEffect* function in limma was deemed to have minimal sequencing batch effects after a visual inspection of PCA and used for all downstream analyses. Raw data were normalized using the trimmed mean of M-values (TMM) normalization. To minimize heteroscedasticity from the count data and incorporate precision weights to account for the mean-variance relationship, the *voom* function was applied on the normalized data [60]. A linear model was fit on the voom-normalized data, while adjusting for the study arm (placebo vs metformin) and biopsy time (baseline, 14 weeks of PRT with treatment and young). Due to the paired nature of the study, the participant id was used as a blocking variable. Differential gene expression was measured using an Empirical Bayes statistic in limma for the following comparisons –plaPRT vs Baseline, metPRT vs Baseline, Young vs Old Baseline, Young vs plaPRT (16 weeks), and Young vs metPRT (16 weeks). The raw P-values were adjusted for multiple comparisons using the Benjamini-Hochberg correction. The adjusted P-value threshold of 0.01 was used to characterize statistically significant differentially expressed genes (DEG).

### Pathway overrepresentation analysis

Genes that were deemed to be differentially expressed with statistical significance (FDR-adj P-value < 0.01), were exported to ConsensusPathdb (http://cpdb.molgen.mpg.de/CPDB) database [61]. The pathways interrogated were Reactome, KEGG, Biocarta, Wikipathways and PharmGKB. A hypergeometric test was run and pathways with a gene overlap threshold of 10% of all input genes and the p-value cutoff of 0.01 were included in the output. Duplicated pathways were excluded from the output.

### Data availability statement

The raw RNA-Seq and count data from this experiment will be accessible in the Gene Expression Omnibus database (GEO Accession code- GSE157585).

## Supplementary Material

Supplementary Table 1

Supplementary Table 2

Supplementary Tables 3 and 4

Supplementary Table 5

Supplementary Table 6

Supplementary Table 7
