# Supplementary material for: Metformin alters skeletal muscle transcriptome adaptations to resistance training in older adults
Source: Aging (Albany NY). 2020 Oct 18;12(20):19852–66. doi: 10.18632/aging.104096 (PMC7655218; doi:10.18632/aging.104096)
Supplement: Supplementary Tables 3 and 4 [file aging-12-104096-s003..pdf]

SUPPLEMENTARY TABLES

Supplementary Table 3. List of aging-associated pathways identified to be overrepresented in the Differentially Expressed Genes unique to 16 weeks of metPRT (and not plaPRT) vs baseline.

Pathways	p-value	q-value	Source	Overlap with Differentially Expressed Genes
Cellular senescence	1.48E-05	0.0009203	KEGG	MAPK14; NFATC3; E2F1; RHEB; GADD45A; LIN37; CCNE1; SERPINE1; RRAS; TGFB3; PIK3R3; VDAC1; ITPR1; RAF1; MAPKAPK2
Platelet activation, signaling and aggregation	0.00012627	0.00391438	Reactome	MAPK14; TGFB3; ITPR1; RAC1; GNB4; MAGED2; VAV2; SERPINE1; HABP4; FERMT3; GNA14; PIK3R3; LAMP2; VTI1B; ITIH3; TOR4A; RAF1; ITIH4
Autophagy	0.00043644	0.00901974	KEGG	RAB7A; RHEB; SUPT20H; ATG16L1; RRAS; BAD; PIK3R3; LAMP2; ATG9A; ITPR1; RAF1
Platelet degranulation	0.00173465	0.01596422	Reactome	TGFB3; MAGED2; ITIH4; HABP4; FERMT3; LAMP2; VTI1B; ITIH3; TOR4A; SERPINE1
Response to elevated platelet cytosolic Ca2+	0.00230046	0.01782854	Reactome	TGFB3; MAGED2; ITIH4; HABP4; FERMT3; LAMP2; VTI1B; ITIH3; TOR4A; SERPINE1
Antigen processing: Ubiquitination and Proteasome degradation	0.00316855	0.01965693	Reactome	KBTBD7; ASB11; UBE2K; ZNRF1; ASB4; LONRF1; ASB5; TRIM32; RNF41; FBXO31; CUL5; UBE2S; RLIM; UBE2R2; FBXL22
Post-translational protein modification	0.0041917	0.02088671	Reactome	ASB11; RAD23B; EEF1A1; CD59; SPON1; PSMD2; RIPK1; ADAMTS13; HIST2H2BE; FOXO4; VDAC1; DCTN1; SAR1B; CCDC8; KBTBD7; RAB34; TTLL3; ASB4; ASB5; XRCC4; PSMA2; GMDS; COPS3; C3; CUL5; TOMM70; NUS1; RAB7A; ST3GAL5; NUP98; UBE2R2; SPARCL1; USP30; F10; ST6GALNAC4; INO80B; EEF1AKMT2; TRAPPC9; UBE2K; WDTC1; ST8SIA2; ARCN1; TRAPPC6A; ADRB2; FBXO31; RNF128; NEU4; RFT1; UBE2S; FEM1A; FBXL22
Neddylation	0.00721409	0.02981822	Reactome	KBTBD7; ASB11; WDTC1; ASB4; ASB5; COPS3; PSMD2; PSMA2; FBXO31; CUL5; CCDC8; FBXL22; FEM1A

Supplementary Table 4. Age and sex of young individuals used in this study.

Study_ID	Age	Sex
CMB-001	23.0	Female
CMB-018	27.2	Female
CMB-020	21.6	Male
CMB-024	28.3	Female
CMB-056	29.7	Male
CMB-065	24.0	Male
CMB-071	25.3	Female
CMB-073	20.7	Female
CMB-079	26.0	Male
CMB-082	24.8	Female
CMB-083	23.5	Male
CMB-087	21.4	Female
CMB-090	22.5	Female
CMB-091	23.5	Male
CMB-099	24.3	Female
CMB-101	25.8	Male
CMB-106	24.3	Male
CMB-107	19.8	Female
CMB-112	21.3	Female
CMB-116	23.9	Male
CMB-123	27.2	Male
