# Supplementary material for: Metformin alters skeletal muscle transcriptome adaptations to resistance training in older adults
Source: Aging (Albany NY). 2020 Oct 18;12(20):19852–66. doi: 10.18632/aging.104096 (PMC7655218; doi:10.18632/aging.104096)
Supplement: Supplementary Table 7 [file aging-12-104096-s006..pdf]

SUPPLEMENTARY TABLE

Supplementary Table 7. List of pathways derived from genes unique to young vs metPRT- 16 weeks that are not differentially expressed compared to young vs baseline.

Pathways	p-value	q-value	Source	Genes Identified as unique to metPRT
Synthesis of UDP-N-acetyl-glucosamine	0.00047005	0.1240602	Reactome	RENBP; GNPNAT1; NAGK
Metabolism of lipids	0.00066041	0.1240602	Reactome	ACP6; RAB4A; SCP2; OSBPL1A; NCOR1; ACER2; GPAM; TECR; LPCAT4; SIN3B; HSD17B7; HMGCS1; RAB5A; OSBPL9; GDE1; OSBPL2; TBL1X; PLA2G4F; PLA2G4E; HACD3; HACD2; FAAH; SC5D; JMJD7-PLA2G4B; PPP1CB; DECR2; PLEKHA8
Cohesin Loading onto Chromatin	0.00097637	0.1240602	Reactome	SMC1A; WAPL; PDS5A
Acyl chain remodelling of PS	0.00121528	0.1240602	Reactome	JMJD7-PLA2G4B; LPCAT4; PLA2G4F; PLA2G4E
Establishment of Sister Chromatid Cohesion	0.0013218	0.1240602	Reactome	SMC1A; WAPL; PDS5A
Hydrolysis of LPC	0.0013218	0.1240602	Reactome	JMJD7-PLA2G4B; PLA2G4F; PLA2G4E
Metabolism	0.00208963	0.16465348	Reactome	OSBPL9; ATIC; RPL9; ATP5S; ACP6; SCP2; GAPDH; UROS; OSBPL1A; NDUFAF7; GSR; SLC25A19; ASS1; SLC25A12; NCOR1; PANK1; GPAM; RAB4A; NT5E; TECR; MOCS2; LPCAT4; GBE1; HMGCS1; NUP210; LDHA; HSD17B7; ACER2; RAB5A; AUH; NUDT3; NUDT5; ETFDH; PHKA2; PLCD4; NUP50; PHGDH; GDE1; OSBPL2; TBL1X; EEF1E1; PLA2G4F; PLA2G4E; HACD3; HACD2; FAAH; SC5D; JMJD7-PLA2G4B; SIN3B; DHODH; PPP1CB; COX15; NAPRT; SQOR; DECR2; XYLB; PLEKHA8; CPS1; COQ2
Biosynthesis of unsaturated fatty acids - Homo sapiens (human)	0.00225553	0.16465348	KEGG	HACD3; HACD2; SCP2; TECR
Acyl chain remodelling of PC	0.00258868	0.16624452	Reactome	JMJD7-PLA2G4B; LPCAT4; PLA2G4F; PLA2G4E
Mitotic Telophase/Cytokinesis	0.00278339	0.16624452	Reactome	SMC1A; WAPL; PDS5A
Acyl chain remodelling of PE	0.00335325	0.17313497	Reactome	JMJD7-PLA2G4B; LPCAT4; PLA2G4F; PLA2G4E
ERBB2 Regulates Cell Motility	0.00342581	0.17313497	Reactome	BTC; MEMO1; EGF
Metabolism of steroids	0.0052267	0.21827294	Reactome	HMGCS1; GPAM; OSBPL1A; SCP2; SC5D; OSBPL9; OSBPL2; HSD17B7
Cardiac Hypertrophic Response	0.00530898	0.21827294	Wikipathways	NFKB1; MTOR; EGF; MAP2K5; MAP2K4
Synthesis of bile acids and bile salts	0.00531563	0.21827294	Reactome	OSBPL2; OSBPL1A; OSBPL9; SCP2
TGF-beta Receptor Signaling	0.00574218	0.22191846	Wikipathways	CTNNB1; SMAD5; NOG; EGF; NFKB1
HSP90 chaperone cycle for steroid	0.00685897	0.23717586	Reactome	HSPA2; NR3C1; FKBP5

hormone receptors (SHR)				
Acyl chain remodelling of PG	0.00685897	0.23717586	Reactome	JMJD7-PLA2G4B; LPCAT4; PLA2G4F
Activation of gene expression by SREBF (SREBP)	0.00794609	0.24669174	Wikipathways	SC5D; GPAM; HMGCS1
Regulation of lipid metabolism by Peroxisome proliferator-activated receptor alpha (PPARalpha)	0.00794609	0.24669174	Reactome	TBL1X; SIN3B; NCOR1
Synthesis of PA	0.00950069	0.24669174	Reactome	JMJD7-PLA2G4B; LPCAT4; GPAM; ACP6
